# *Malva parviflora* Leaves and Fruits Mucilage as Natural Sources of Anti-Inflammatory, Antitussive and Gastro-Protective Agents: A Comparative Study Using Rat Models and Gas Chromatography

**DOI:** 10.3390/ph15040427

**Published:** 2022-03-31

**Authors:** Ahmed E. Altyar, Ans Munir, Saiqa Ishtiaq, Muhammad Rizwan, Khizar Abbas, Osama Kensara, Sameh S. Elhady, Waleed Y. Rizg, Fadia S. Youssef, Mohamed L. Ashour

**Affiliations:** 1 Department of Pharmacy Practice, Faculty of Pharmacy, King Abdulaziz University, P.O. Box 80260, Jeddah 21589, Saudi Arabia; aealtyar@kau.edu.sa; 2 Department of Pharmacognosy, College of Pharmacy, University of the Punjab, Lahore 54000, Pakistan; ansmunir92@gmail.com (A.M.); saiqa.pharmacy@pu.edu.pk (S.I.); 3 Department of Pathology, Lahore Medical and Dental College, University of Health Sciences, Lahore 54600, Pakistan; muhammad.rizwan@lmdc.edu.pk; 4 Department of Pharmacognosy, Faculty of Pharmacy, Bahauddin Zakariya University, Multan 60800, Pakistan; khizarabbas@bzu.edu.pk; 5 Department of Clinical Nutrition, Faculty of Applied Medical Sciences, Umm Al-Qura University, P.O. Box 7067, Makkah 21955, Saudi Arabia; oakensara@uqu.edu.sa; 6 Department of Natural Products, Faculty of Pharmacy, King Abdulaziz University, P.O. Box 80260, Jeddah 21589, Saudi Arabia; ssahmed@kau.edu.sa; 7 Department of Pharmaceutics, Faculty of Pharmacy, King Abdulaziz University, P.O. Box 80260, Jeddah 21589, Saudi Arabia; wrizq@kau.edu.sa; 8 Center of Excellence for Drug Research and Pharmaceutical Industries, King Abdulaziz University, P.O. Box 80200, Jeddah 21589, Saudi Arabia; 9 Department of Pharmacognosy, Faculty of Pharmacy, Ain-Shams University, Abbasia, Cairo 11566, Egypt; fadiayoussef@pharma.asu.edu.eg; 10 Department of Pharmaceutical Sciences, Pharmacy Program, Batterjee Medical College, P.O. Box 6231, Jeddah 21442, Saudi Arabia

**Keywords:** anti-inflammatory, antitussive, cytoprotection, gastric ulcer, *M. parviflora*, mucilage

## Abstract

*Malva parviflora* L., Little mallow, has been traditionally used as an alternative food source. It acts as a medicinal herb containing a potential source of mucilage thus herein; we aimed to assess the toxicity, anti-inflammatory, antitussive and gastro-protective actions of *M. parviflora* mucilage extracted from its leaves (MLM) and fruit (MFM). Toxicity studies were investigated by in vitro hemolytic assay whereas acute anti-inflammatory and antitussive activities were assessed by carrageenan-induced paw edema and sulphur dioxide induced cough model in rats, respectively. Gastro-protective effects were studied using ethanol induced acute and chronic gastric ulcer rat models. Their metabolic profiles were determined using gas chromatography. The results revealed that MLM and MFM were non-toxic towards human erythrocytes and their lethal doses were found to be greater than 5 g/kg. Pretreatment with MLM (500 mg/kg) and MFM (500 mg/kg) significantly reduced the carrageenan-induced paw thickness (*p* < 0.001). Maximum edema inhibition (%) was observed at 4 h in diclofenac sodium (39.31%) followed by MLM (27.35%) and MFM (15.68%). Animals pretreated with MLM (500 mg/kg) significantly lower the cough frequency in SO_2_ gas induced cough models in contrast to control. Moreover, MLM at doses of 250 and 500 mg/kg reduced the ethanol induced gastric mucosal injuries in acute gastric ulcer models presenting ulcer inhibition of 23.04 and 38.74%, respectively. The chronic gastric ulcer model MFM (500 mg/kg) demonstrated a remarkable gastro-protective effect showing 63.52% ulcer inhibition and results were closely related to standard drug sucralfate. In both models, MLM and MFM decreased gastric juice volume and total acidity in addition to an increased gastric juice pH and gastric mucous content justifying an anti-secretary role of this mucilage that was further confirmed by histopathological examination. Meanwhile, GC analyses of the mucilage revealed their richness with natural as well as acidic monosaccharides. It is concluded that MLM and MFM can be used therapeutically for the management of inflammation, cough and gastric ulcer.

## 1. Introduction

Demulcents form a protective coating over mucous membranes alleviating pain and inflammation becoming slimy upon contact water [1]. They are indicated to heal hot, irritated, dry and inflamed mucous membranes and for ulcers, sore throat, inflammation in bowels and upper respiratory tract, and for bladder infections [2]. They can also act as prebiotics and normalize intestinal flora population. They repair the mucosa by reducing the irritation of bowel and decreasing sensitivity to gastric acid [3]. Histological studies have revealed that consumption of demulcent herbs results in the presence of polysaccharides layers on the membrane surface [4].

Demulcents are medicinally important as they contain high amounts of mucilage. Plant mucilage is a gelatinous substance chiefly comprising polysaccharides and uronic acid, as well as glycoproteins with other biologically active substances including tannins, alkaloids and steroids [5,6]. Mucilage is a renewable and inexpensive source of non-toxic, bioactive and eco-friendly compounds and have extensive applications in pharmaceutical, food and nutraceutical, cosmetics, textile, paper and paint industries [7]. The biologically active and nutraceutical characteristics, along with their possible health blessings, mark mucilage as an important ingredient in a healthy diet. They have been used as an antioxidant, antidiabetic, anticancer, antifungal, antimicrobial, anti-inflammatory, wound healer, ACE enzyme inhibitor, hypolipidemic agent and immune-stimulator. Further, they are used to treat skin, gastrointestinal, respiratory and urinary disorders [8,9]. It is worthy to highlight that a number of plant mucilages are available in the literature that have been approved by FDA for their additive roles in food, cosmetics and pharmaceutical industries i.e., Fenugreek seed mucilage, *Hibiscus rosa-sinensis* mucilage *Lepidium sativum* mucilage, *Aloe vera*, *Plantago ovata* seed mucilage [10].

Moreover, inflammation is the first response of body’s immune system to stress, injury and infection. Prolonged and persistent inflammation could be harmful leading to the development of diseases such as fever, asthma, arthritis, atherosclerosis, auto-immune disorders and cancer [11,12,13]. The treatment of inflammation usually depends on steroidal and non-steroidal anti-inflammatory drugs that reveal side effects such as gastrointestinal ulcer, hypertension, osteoporosis, hepatotoxicity, kidney disorders as well as allergies [14]. Gastric ulcers are a long-lasting illness affecting thousands of people globally. This not only disturbs everyday life of affected individuals but is also sometimes accompanied by fatal complications i.e., gastric bleeding and perforations [15].

Traditionally, natural products were utilized to cure and manage stomach ulcers [16,17,18]. They proposed effective, inexpensive and easily available forms of treatment for individuals affected by gastric ulcers [19]. Thus, unveiling of the most active and safe ulcer healing and protective agent from natural products is of great value [20]. This suggests that seeking new natural anti-inflammatory and gastro-protective agents with limited side effects is needed for human health [21]. Furthermore, mucilage polysaccharides obtained from different plants are used in children with acute cough due to their emollient and demulcent properties. Their mucus-protective and bio-adhesive properties are useful in oral and gastric disorders [22].

Little mallow plant (*Malva parviflora* L.) are among the edible crops and have been part of the Mediterranean diet for a long time [23]. It is generally recognized as cheese-weed, small whorl mallow and in Pakistan as Sonchal. Traditionally decoction of the entire plant has been used as a remedy for fever, cold and cough meanwhile its leaves are used as a vegetable and an emollient. They are used to treat inflammation, wounds, gastritis, bladder ulcers, diuretic, constipation, abdominal pain, diarrhea, anthelmintic, hair loss, ocular disease, scorpion sting and profuse menstruation [24]. Immature fruits are used as snacks. Mallows have a long history of medicinal use due to their high antioxidant activity and anti-inflammatory potential. Pharmacologically, *M. parviflora* have been evaluated for its antioxidant, anti-inflammatory, antimicrobial, antiulcer, antidiabetic, anti-irritant, hepatoprotective, neuroprotective, analgesic and wound healing properties [25]. Although anti-inflammatory and gastro-protective effects of crude methanol and ethanol extracts of *M. parviflora* leaves have been reported, no research was found in the literature regarding such activities of *M. parviflora* leaves and fruit mucilage [26,27,28,29].

Hence, our previous study reported the potent antioxidant, DNA damage and skin protection activity of *M. parviflora* leaves mucilage [25], thus, herein our main objective was to compare both *M. parviflora* leaves and fruit mucilage in virtue of their toxicity, anti-inflammatory, antitussive and gastro-protective activities aiming to use them as potential food sources to alleviate many health disorders. In vitro and in vivo toxicity was studied by hemolytic assay and following the OECD 425 guidelines, respectively. Acute antitussive and anti-inflammatory activities were investigated using sulphur dioxide induced cough models and carrageenan-induced paw edema models in rats, respectively. In addition, ethanol induced acute and chronic gastric ulcer models were used to study the gastro-protective effects of *M. parviflora* leaves (MLM) and fruit (MFM) mucilage. Moreover, their comparative metabolic profiles were determined using gas chromatography. Meanwhile, it is noteworthy to mention that this is the first study to report the toxicity and antitussive activity of *M. parviflora* leaves and fruit mucilage. Previously, no data were available regarding *M. parviflora* mucilage, meanwhile the clinical role of mucilage is an emerging research area and remains still under investigation.

## 2. Results

### 2.1. In Vitro Hemolytic Activity

**Figure 1:**
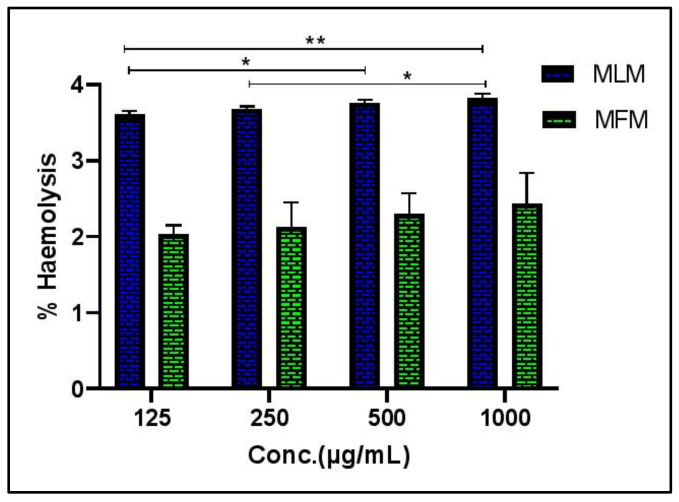
Hemolytic activity of MLM and MFM at different concentrations, 125, 250, 500 and 1000 μg/mL; PBS (negative control) showed no hemolysis whereas Triton-X (positive control) showed 100% hemolysis; Significant at *p* < 0.05 * and 0.01 **.

Hemolysis assay is suitable to investigate whether cytotoxicity is associated with direct damage to cell membrane or not. Hemolytic activity of MLM and MFM was screened against human erythrocytes at four different concentrations which are 125, 250, 500 and 1000 µg/mL. Full hemolysis (100%) was attained using 0.1% Triton X-100 whereas no hemolysis was observed with Phosphate-buffered saline (PBS). Both mucilages displayed little hemolytic result towards human RBCs. Very weak hemolysis was observed for MLM and MFM even at high concentrations showing 3.81 and 2.43% hemolysis at 1000 µg/mL respectively. Results for hemolytic activity as depicted in Figure 1 supports the safety of MLM and MFM towards human erythrocytes.

### 2.2. Acute Toxicity Study

**Figure 2:**
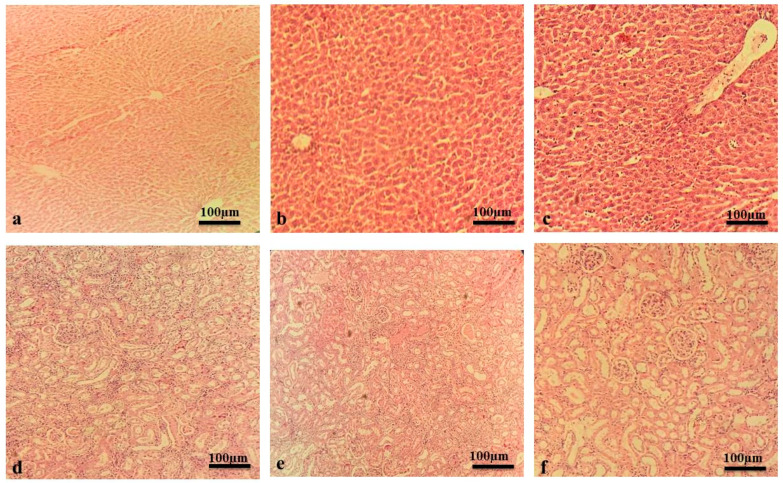
Liver and kidney histology from acute toxicity assay. (**a**) Liver of vehicle control group. (**b**) Liver of MLM-treated group. (**c**) Liver of MFM-treated group. (**d**) Kidney of vehicle control group. (**e**) Kidney of MLM-treated group. (**f**) Kidney of MFM-treated group.

In acute toxicity studies, all rats remained alive showing no signs of toxicity or abnormalities observed at a dose of 5 g/kg of MLM and MFM, administered orally. For the period of fourteen days, there were no signs of abnormalities evidenced by no behavioral changes or alteration in body weight. There was a non-significant difference in serum biochemical parameters of treated group and control group (Table 1). Histological analysis showed no signs of hepatic or renal toxicity as compared to control group (Figure 2). Thus, it was concluded that the oral lethal dose for MLM and MFM would be greater than 5 g/kg. These results support the safety of MLM and MFM even at high dose.

### 2.3. Acute Anti-Inflammatory Activity

**Figure 3:**
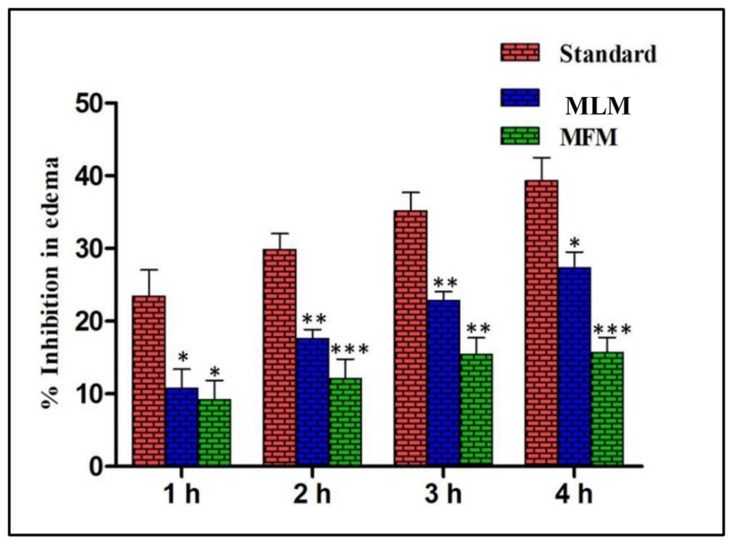
Percentage inhibition of edema by MLM and MFM (500 mg/kg) at different time intervals; Significant at *p* < 0.05 *, 0.01 ** and 0.001 ***.

The carrageenan-induced paw edema model was carried out to investigate the anti-inflammatory potential of mucilage. Sub plantar injection of 1% carrageenan (0.1 mL) to rat hind paw steadily increased the paw thickness that extends to maximum after 4 h. Pretreatment with MLM (500 mg/kg) and MFM (500 mg/kg) significantly reduced the paw thickness with maximum inhibition at 4 h as compared to the disease control group (*p* < 0.001). MLM exhibited better inhibition of paw thickness as compared to MFM but not as much as diclofenac sodium (Table 2). The maximum percentage of anti-inflammatory activity was observed in diclofenac sodium (39.31%) at 4 h followed by MLM (27.35%) and MFM (15.68%) as depicted in Figure 3. Percentage inhibition of edema observed by MLM was significantly comparable with diclofenac sodium (*p* < 0.05).

### 2.4. Acute Antitussive Activity

**Figure 4:**
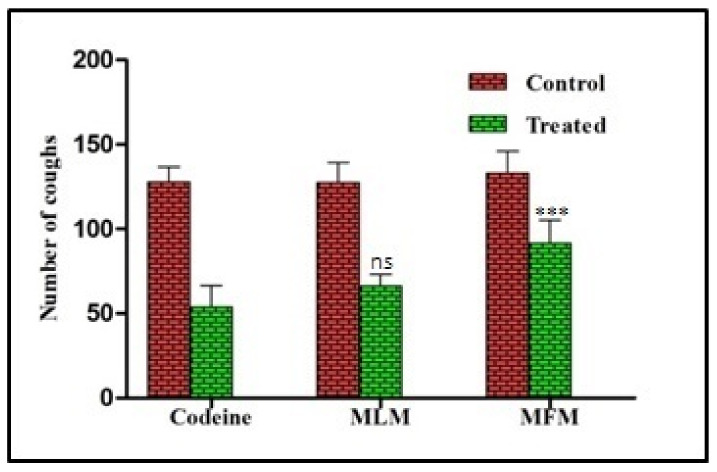
Number of coughs in control and treated groups with codeine phosphate (10 mg/kg), MLM (500 mg/kg) and MFM (500 mg/kg); Significant at *p* < 0.001 ***, ns = not significant.

Codeine (10 mg/kg) reduced cough frequency from 127.6 to 53.8; marking a 57.8% inhibition in the incidence of cough. Oral administration of 500 mg/kg of MLM significantly decreased the number of coughs induced by SO_2_ gas from 127.4 to 66.2; marking a 47.99 % inhibition in the incidence of cough. The least antitussive activity was observed in animals treated with 500 mg/kg of MFM that reduced the cough frequency from 133 to 91.66 with 31.37% inhibition of cough (Figure 4). There was a non-significant difference in percentage inhibition of cough in MLM treated rats when compared with standard drug.

### 2.5. Antiulcer Activity

#### 2.5.1. Macroscopic Analysis of Stomach Mucosa

**Figure 5:**
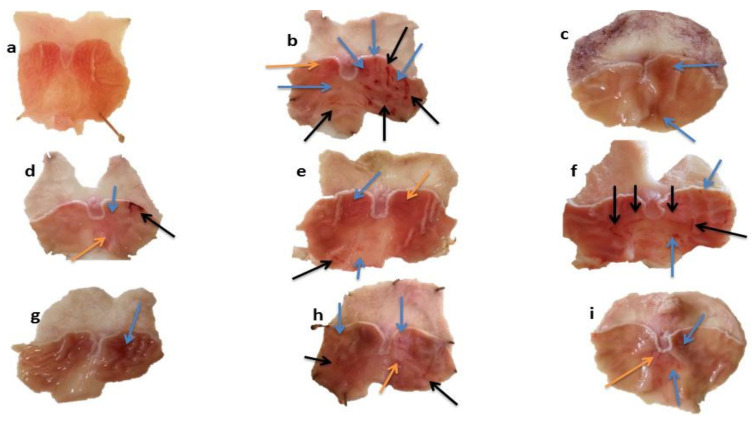
Macroscopic view of gastric mucosa in acute gastric ulcer model of (**a**) Normal (**b**) Ethanol (**c**) Omeprazole (**d**) Ranitidine (**e**) Sucralfate (**f**) MLM 250 mg/kg (**g**) MLM 500 mg/kg (**h**) MFM 250 mg/kg (**i**) MFM 500 mg/kg groups. Black arrows = hemorrhagic streaks, Blue arrows = Ulcer spots, Orange arrows = reddish mucosa.

The stomach walls of each rat was observed by the naked eye and then by hand lens. Macroscopic view of gastric walls of normal group showed smooth surface without noticeable scars. In acute gastric ulcer models, severe lesions with extensive visible hemorrhagic streaks were observed in the ulcer control group. Moderate lesions were seen in rats pretreated with MLM (250 mg/kg) and MFM (250 mg/kg and 500 mg/kg). Low moderate lesions were seen in rats pretreated with ranitidine, sucralfate and MLM (500 mg/kg). However, mild lesions were observed in omeprazole treated group which directs high protection against gastric ulcer (Figure 5).

**Figure 6:**
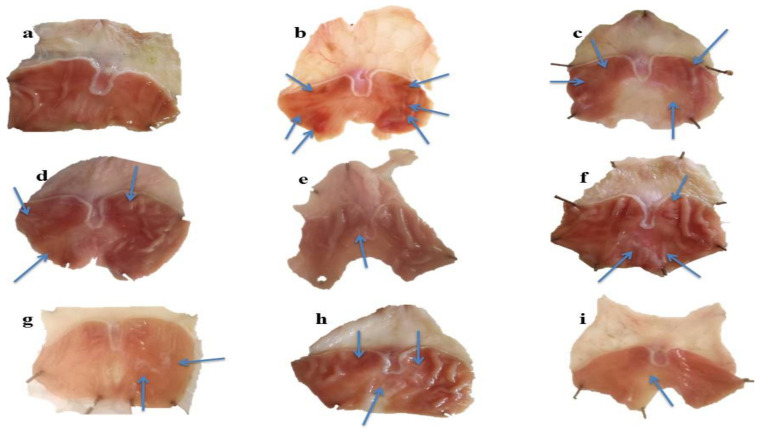
Macroscopic view of gastric mucosa in chronic gastric ulcer model of (**a**) Normal (**b**) Ethanol (**c**) Omeprazole (**d**) Ranitidine (**e**) Sucralfate (**f**) MLM 250 mg/kg (**g**) MLM 500 mg/kg (**h**) MFM 250 mg/kg (**i**) MFM 500 mg/kg groups. Blue arrows = Ulcer spots.

In chronic gastric ulcer models, ethanol induced gastric ulcer scars and lesions that were visible, but the nature of lesions was different as compared to the acute ulcer models. The lesions are generally categorized by the existence of white rounded scars with rare hemorrhage. Moderate scars were present in gastric mucosa of rats pretreated with ranitidine, omeprazole and MLM (250 mg/kg). However, rare scars were observed in rates pretreated with sucralfate, MLM (500 mg/kg) and MFM (Figure 6).

#### 2.5.2. Determination of Ulcer Score, Ulcer Index and Percentage Inhibition

The ulcer index (UI) and % ulcer inhibition of experimental and control groups in acute gastric ulcer are given in Table 3. UI for the ethanol group was 11.36 meanwhile omeprazole, ranitidine and sucralfate considerably reduced the UI to 3.46, 5.21 and 5.27 with ulcer protection of 69.56%, 54.21% and 53.63%, respectively, where omeprazole showed maximum ulcer protection. Pretreatment with MLM at dosage of 250 mg/kg and 500 mg/kg decreased the UI to 8.74 and 6.96 and gave ulcer protection of 23.04% and 38.74%, respectively. Pretreatment with MFM at dosage of 250 mg/kg and 500 mg/kg decreased the UI to 10.47 and 7.03 and gave ulcer protection 7.85% and 38.08%, respectively. Thus, it was clear that pretreatment with MFM and MLM resulted in an increased ulcer inhibition activity in a dose–dependent manner (Table 3).

Meanwhile, in chronic gastric ulcer model, UI of ulcer control group, ethanol group, was 10.8. Pretreatment with omeprazole, ranitidine and sucralfate considerably reduced the UI to 5.22, 3.5 and 3.51 showing ulcer protection of 51.66, 67.59 and 67.5%, respectively. As compared to acute ulcer model, in chronic gastric ulcer model ranitidine and sucralfate showed maximum ulcer protection estimated by 3.5 and 3.51 accounting for 67.59 and 67.50%. Pretreatment with MLM at dosages of 250 mg/kg and 500 mg/kg decreased the UI to 7.08 and 5.67 and gave ulcer protection of 34.44 and 47.44%, respectively. Pretreatment with MFM at dosages of 250 mg/kg and 500 mg/kg decreased the UI to 6.95 and 3.95 and gave ulcer protection of 35.65 and 63.52%, respectively (Table 4). As observed in acute gastric ulcer models, dose–dependent ulcer inhibition was also observed in chronic gastric ulcer model; however, in chronic ulcer MFM was found to be more effective in contrast to acute gastric ulcer models where MLM revealed higher efficacy.

#### 2.5.3. Histology of Stomach Wall

**Figure 7:**
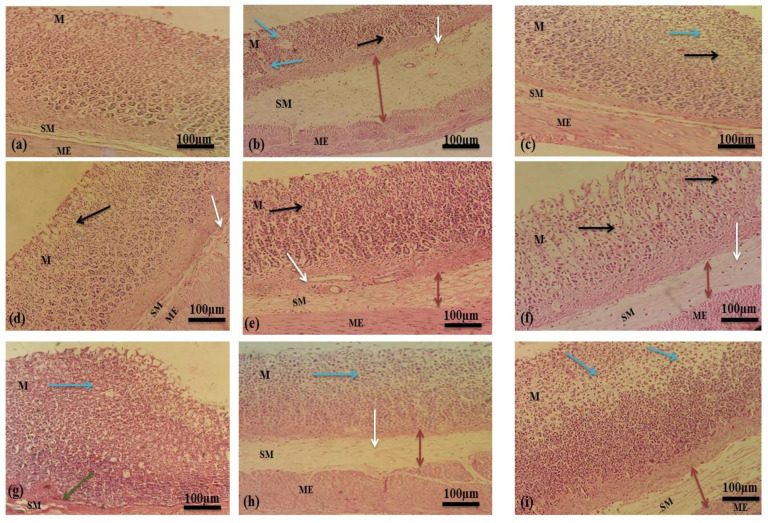
Histological evaluations in acute ulcer model (**a**) Normal control group (**b**) Ethanol (Ulcer control group) (**c**) Omeprazole (**d**) Ranitidine (**e**) Sucralfate (**f**) MLM 250 mg/kg (**g**) MLM 500 mg/kg (**h**) MFM 250 mg/kg (**i**) MFM 500 mg/kg. M represents mucosa, SM represents submucosa and ME represent muscularis externa. Blue arrows indicate areas of ulceration and focal erosion. Black arrows indicate the mucosal edema. Dark red double headed arrows indicate submucosal edema. White arrows indicate leukocyte infiltration. Green arrows indicate congestions.

**Figure 8:**
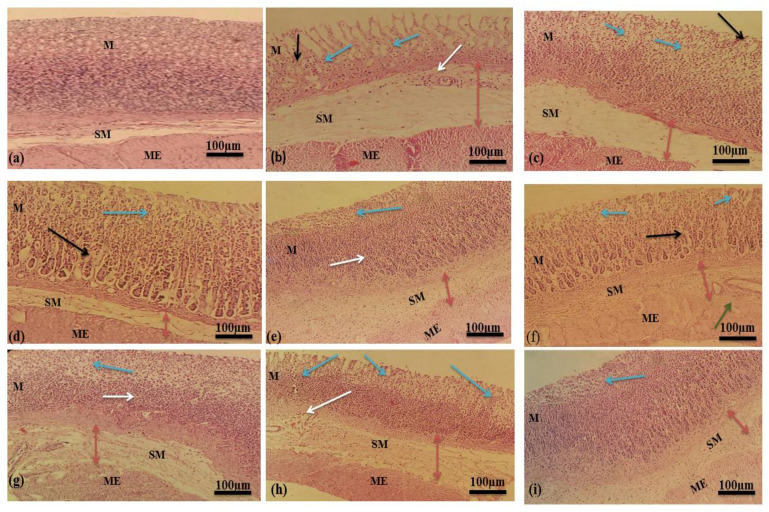
Histological evaluations in chronic ulcer model (**a**) Normal control group (**b**) Ethanol (Ulcer control group) (**c**) Omeprazole (**d**) Ranitidine (**e**) Sucralfate (**f**) MLM 250 mg/kg (**g**) MLM 500 mg/kg (**h**) MFM 250 mg/kg (**i**) MFM 500 mg/kg. M represents mucosa, SM represents submucosa and ME represent muscularis externa. Blue arrows indicate areas of ulceration and focal erosion. Black arrows indicate the mucosal edema. Dark red double headed arrows indicate submucosal edema. White arrows indicate leukocyte infiltration. Green arrows indicate congestions.

Histological examination of gastric mucosa revealed that ethanol caused severe disruption in the gastric mucosa that pierced deeply, accompanied by mucosal and sub-mucosal edema and leukocyte infiltration. Thickness of mucosal layer decreased extensively in chronic ulcer models due to the daily interruption of ethanol with gastric mucosa. The standard drugs and mucilage established enhanced protection of gastric mucosa in a dose–dependent manner accompanied by a pronounced reduction in edema and leukocytes infiltration of sub-mucosal layers. Histological observations in acute and chronic ulcer model are shown in Figure 7 and Figure 8.

#### 2.5.4. Mechanism of Action

##### Effect on Gastric Juice Parameters

**Figure 9:**
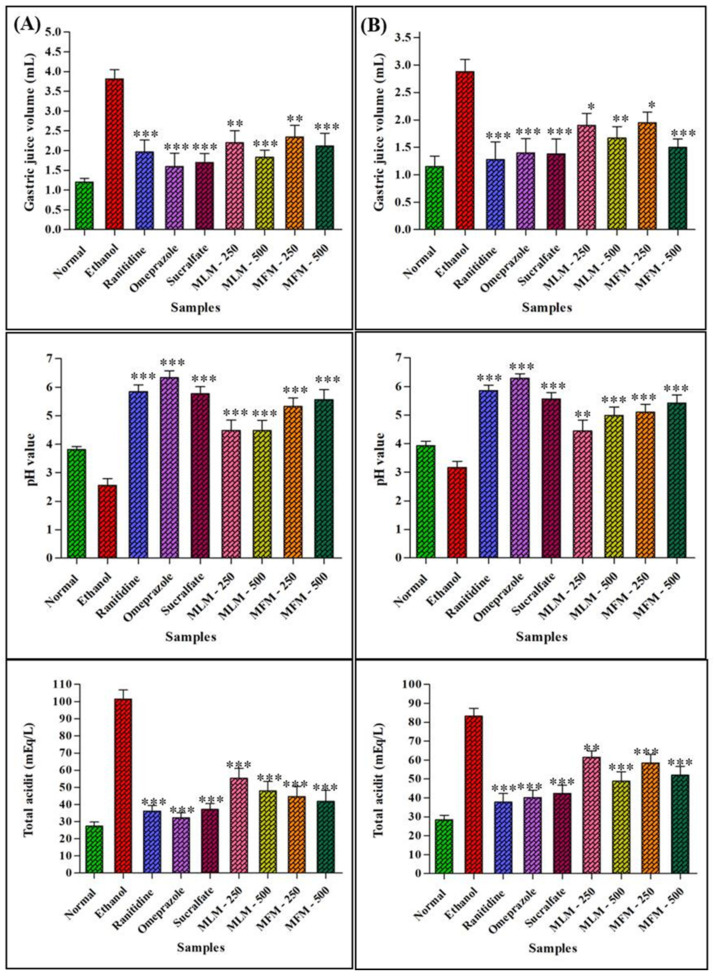
Effect of MLM and MFM on gastric juice volume, pH and total acidity in acute (**A**) and chronic ulcer (**B**); The results are expressed in the form of Mean ± SEM. Significant at *p* < 0.05 *, 0.01 ** and 0.001 ***.

Gastric juice of ulcer control group displayed higher gastric juice volume, lower pH and high total acidity in contrast to normal animals. Pretreatment with MLM (500 mg/kg) and MFM (500 mg/kg) significantly (*p* < 0.001) reduced the gastric juice volume and total acidity but increased pH when compared with ulcer control group as illustrated in Figure 9. Similar changes were also observed in chronic gastric ulcer models (Table 5).

##### Effect on Total Protein and Total Mucus Content

The Alcian blue binding capacity was used as a marker for quantification of gastric mucus content. Ethanol treatment reduced the total gastric mucus content and ultimately Alcian blue binding capacity. Pretreatment with MLM (500 mg/kg) and MFM (500 mg/kg) in acute gastric ulcer models significantly (*p* < 0.01) protected the gastric mucosa increasing the mucus content in contrast to the ulcer control group. However, at low dosages (250 mg/kg), a non-significant difference was observed in total mucus content of MLM and MFM with ulcer control group. Ranitidine and MLM (500 mg/kg) had total protein content significantly dissimilar from ulcer control group (*p* < 0.01). However, in all other treated group a non-significant difference was observed in comparison to ulcer control group. In chronic gastric ulcers, there was a dramatic rise of total mucous content in animals pretreated with MLM (500 mg/kg), MFM (500 mg/kg), MFM (250 mg/kg) and sucralfate (100 mg/kg) significantly different from ulcer control group (*p* < 0.001). A non-significant difference was observed between total mucus content of animals pretreated with MLM (250 mg/kg) and ranitidine when compared with ulcer control group. Total protein content was also significantly different in the treated group as compared to the ulcer control group (Table 5).

### 2.6. Gas Chromatography Analyses of MLM and MFM

GC/MS analysis of MFM revealed the existence of four neutral monosaccharides which are galactose, rhamnose, arabinose as well as glucose constituting about 50.21, 7.61, 6.18 1.90 mg/g of MFM, respectively, whereas galacturonic acid constitutes the identified acidic monosaccharide showing 16.02 mg/g of MFM (Figure S1). Regarding the main monosaccharides detected in MLM, they are previously analyzed by the authors and reported [25] where GC/MS analysis of MLM showed the presence of five neutral monosaccharides together with one acidic monosaccharide which are galactose, rhamnose, arabinose, mannose as well as glucose and galacturonic acid accounting for 51.09, 10.24, 8.90, 1.80, 0.90 and 15.06 mg/g of MLM, respectively. Results showed that the leaves mucilage showed slightly higher levels of neutral monosaccharides compared to the fruit mucilage in contrast to the acidic monosaccharide represented by galacturonic acid that displayed a higher level in the fruit mucilage.

## 3. Discussion

Plant derived natural therapeutic agents have become part of primary healthcare in developing countries. Because of their natural origin some of them are mistakenly considered as absolute safe drugs. Thus, there is a great need of scientific studies on toxicities of drugs obtained from natural sources [30]. Erythrocytes are abundantly present in the human body, and they show vast biological and structural attributes playing an important role in drug transport. Breakdown of erythrocytes is known as hemolysis that occurs upon exposure of erythrocytes to toxicants. Some phytochemicals can cause hemolysis that in turn reflects that given substance is cytotoxic to RBCs [31,32].

Erythrocyte membranes are made up of polyunsaturated fatty acids and proteins, therefore, more vulnerable to peroxidation. Hemoglobin present in RBCs also catalyzes the oxidation process. Oxidation of RBCs represents oxidative damage of other biological membranes. Thus, the chemicals which produced free radicals can damage the erythrocyte membrane and serve as hemolytic agent [33]. In vitro hemolytic activity of MLM and MFM was assessed in an effort to check their toxicity to red blood cells. The results clearly justified the safety of MLM and MFM for RBCs and hence, can be used for further in vivo studies. Furthermore, acute oral toxicity of extracted mucilage was also performed to identify the dose that can be safely used for *in-vivo* studies. It was concluded that MLM and MFM are totally safe for systemic use and lethal dose is above 5 g/kg. As 5 g/kg showed no signs of toxicities therefore 1/20 and 1/10 of this lethal dose were selected in the current study (250 mg/kg and 500 mg/kg, respectively) for in vivo studies.

Regarding inflammation, it is defined as a localized reaction accompanied by soreness, warmth, swelling, tenderness and loss of function. Inflammatory mediators are released upon exposure to inflammatory agents. These inflammatory mediators widen the blood vessels and cause chemotaxis and ultimately lead to many disorders [34]. Red seaweeds (Rhodophyceae) are the source of carrageenan which is a sulphated polyglactan with esterified sulphate group that is responsible for its chemical activity. Carrageenan-induced paw edema is the most often practiced model for acute anti-inflammatory activity [34].

Carrageenan generates oxygen free radicals by changing the neutrophil membrane. Neutrophils are responsible for acute external inflammation and rapidly move towards the site of inflammation. They engulfed the pathogens and trashes of damaged tissues and consequently produce the reactive oxygen species and proteolytic enzyme to tear down the engulfed particles. Host tissues are also damaged during this process. As a result of these activities, a deficiency of ATP occurs resulting in the loss of cell function ultimately leading to necrosis [35]. The current study determined the anti-inflammatory activity of mucilage. The results showed that maximum edema inhibition occurred at the fourth hour by diclofenac sodium followed by MLM and MFM where MLM showed better anti-inflammatory activity when compared to MFM. Anti-inflammatory activity of MLM increased with increase in time with maximum results at the fourth hour. This might be due to the radical scavenging effects of MLM and MFM against free radicals produced at site of inflammation. The polysaccharides present in this mucilage may be the active ingredient against inflammation. Previously, mucilage extracted from lemon demonstrated pronounced anti-inflammatory activity in carrageenan-induced paw edema model in rats [35]. Okra and Baobab belongs to the family Malvaceae and their mucilage polysaccharides have been reported to possess anti-inflammatory properties [36].

Concerning the antitussive activity, there are various causes of cough that can be induced by any type of irritation to bronchi and trachea. This irritation can be the result of exposure to light, allergens, chemicals or any other foreign matter. As a result, airway sensory nerves are activated followed by increased mucous secretion, release of inflammatory mediators and damage to airway epithelium. Antitussives suppress cough either by acting centrally or peripherally. Centrally acting antitussive agents suppress the cough center in the brain and peripherally acting agents work as a demulcent or local anesthetic [37]. In this study, SO_2_ gas was used as an irritant to induce cough in rats. SO_2_ was produced by chemical reaction between sodium hydrogen sulphite and concentrated sulphuric acid. As all conditions were kept the same, it was therefore supposed that the quantity and saturation of SO_2_ would remain same during each exposure. Therefore, quantification of SO_2_ was not performed. The results showed that MLM exhibited 47.99% inhibition against chemical-induced cough. MLM was a better cough suppressant than MFM thus supporting the traditional use of leaves of *M. parviflora* as an antitussive. MLM and MFM might act peripherally due to the demulcent properties of mucilage. Folk use of *M. parviflora* is as a demulcent with mucilage responsible for this demulcent action [38]. Hence, this study consolidated the demulcent action of mucilage of *M. parviflora* based on scientific evidence. Rhamnogalacturonan was previously isolated from Marshmallow (*Althea officinalis*) mucilage that also belongs to family Malvaceae and showed high cough suppressant action in guinea pigs [39]. Similarly in an open trial, patients suffering from cough were treated successfully with herbal cough syrup containing marshmallow root mucilage [40].

Gastric ulcer is the chief health issue of GIT where gastric acid, pepsin and *H. pylori* are among the destructive factors that aggravate gastric ulcers, meanwhile the protective factors include mucin, NO, bicarbonates, blood flow, prostaglandins and growth factors. When a disturbance occurs between destructive and defensive factors, gastric ulcers occur [41]. The mucosal layer of stomach releases mucous that contains glycoprotein and lipids. Aside from suppressing the oxygen-derived free radicals it stimulates smooth movement of food and protects the stomach against the damaging effects of HCl and pepsin by preventing their penetration in the mucosal membrane [42,43]. Ethanol causes gastric ulcers as it solubilizes the mucous and diffuses into gastric mucosa. As a result, HCl and pepsin come in contact with gastric mucosa and injure it, in addition, ethanol increases gastric acid secretion. Moreover, it ruptures vascular endothelium, increases vascular permeability, increases oxidative stress in tissue and decreases gastric mucous secretion and thus ethanol induced gastric ulcer models were found to be suitable [44].

Hence, the antiulcer activities of MLM and MFM were evaluated in ethanol induced acute and chronic gastric ulcer models. It has been reported that antacids, anti-secretory agents and cytoprotective agents are the main classes of drugs used for the management of ulcers [45]. Therefore, in this study omeprazole, ranitidine and sucralfate were used as standard drugs. The results showed that *M. parviflora* mucilage and standard drugs significantly reduced the ulcer count and ulcer index with increased cytoprotective effects in both acute and chronic models, whereas the ulcer control group presented severe hemorrhagic streaks. The necrotic and petechial lesions in gastric mucosa leading to gastric ulcers are attributed to ethanol treatment [43]. Extreme stomach mucosa disruption leads to lower release of bicarbonate ions that in turn lowers the pH via increased production of gastric contents. Hence, ethanol increases gastric acid secretion that may result in increased gastric volume, decreased pH, increased total acidity and increased ulcer index [46]. A significant increase in pH and reduction in gastric volume and acidity was noticed in rats pretreated with MLM, MFM and standard drugs when compared with ulcer control groups as represented in Figure 9. This predicted the anti-secretory and antacid mechanism of the gastroprotective effects of MLM and MFM.

In addition, a reduced amount of protein content in stomach tissue homogenate is marked as a sign of damage to normal cellular functions. Therefore, treatments that cause an increase in protein content may be considered to contain auto-healing agents that support the mucosal regeneration process [40]. The low protein content in the disease control group of acute and chronic models was a sign of cellular dysfunction. The protein content in experimental and standard groups was higher compared with the disease control group thus supporting the presence of auto-healing effects. Treatment with MLM and MFM augmented the regeneration of epithelial cells and thus considerably amplified the protein concentration. Meanwhile, gastric mucus also acts as a shield for the protection of gastric walls against aggressive factors and as a first line of mucosal protection from luminal acid [47]. It contains viscid, flexible and translucent gel composed of glycoproteins (5%) and water (95%) and can be identified by the extent of Alcian blue binding. The defensive role of the mucus shield relies on the structure as well as the density of the layer protecting the mucosal surface. Furthermore, mucus can also act as an antioxidant and can diminish free radical-induced mucosal damage [48]. Administration of MLM and MFM significantly raised the amount of mucous in contrast to the disease control group. MLM and MFM treatment increased the gastric mucous content directly dependent on dosage. Interestingly, production of mucus in chronic ulcer models have remarkably been increased. The highest mucus content was observed in rats pretreated with MFM (500 mg/kg) followed by MLM (500 mg/kg) and results were comparable with rats pretreated with sucralfate. This unusual increase in gastric mucus suggested that MLM and MFM may trigger the discharge of chemical mediators responsible for the gastric mucus production i.e., gastrin, prostaglandin, secretin and acetylcholine [49]. Hence, it can be predicted that an increased secretion of mucus by administering MLM and MFM may be one of the potential mechanisms of their gastroprotective action. Mucilage contains polysaccharides where previous studies reported that polysaccharides possess antiulcer activity. These mucilaginous polysaccharides may form a protective covering on gastric mucosa or regenerate it [19]. This can be justified by the high mucus content in both acute and chronic gastric ulcer models.

## 4. Materials and Methods

### 4.1. Plant Material

*Malva parviflora* was collected in February 2018 from Lahore, Punjab, Pakistan. Plant Taxonomist, Dr. Zaheer-ud-din khan at Department of Botany, Government College University, Lahore authenticated the plant and issued a voucher specimen number GC.Herb.Bot.3533. Dusty material was removed by washing the leaves and unripe fruits with tap water. Leaves were dried in shade and concomitantly powdered in a mechanical grinder. The powdered leaves and fresh unripe fruits were used for the extraction. The mucilage was dried in an oven at 45 °C till it was completely dried. The dried mucilage was then powdered by mortar and pestle. The powdered mucilage was passed through a sieve#80. Particle size of the obtained powder was uniform [50,51,52].

### 4.2. Drugs and Chemicals

Omeprazole, sucralfate and ranitidine were used as standard anti-ulcer drugs and were kindly provided by Schazoo Zaka (Pvt.) Ltd. (Sheikhupura, Punjab, Pakistan), Highnoon Laboratories (Pvt.) Ltd. (Lahore, Punjab, Pakistan) and Surge Laboratories (Pvt.) Ltd. (Sheikhupura, Punjab, Pakistan), respectively. Alcian blue dye was obtained from Unichem, china, bovine serum albumin from Bioshop (Burlington, Ontario, Canada) and normal saline from Otsuka (Karachi, Pakistan. Diethyl ether and sucrose were acquired from Labscan (Bangkok, Thailand). Sodium hydrogen sulphite, sodium hydroxide, Sodium carbonate, potassium chloride, Tris HCl and phenolphthalein were purchased from BDH chemicals (Poole, UK). All other chemicals used in this study were obtained from Sigma-Aldrich (Steinheim, Germany). All the reagents were prepared freshly and were of pharmaceutical grade.

### 4.3. Animals

Wistar albino rats of both sexes (180–200 g) were maintained under standard conditions at (22–24 °C). The relative humidity of 50–60% and photoperiod of 12 h light and 12 h dark cycle was retained. Animals were fed on commercial pellet food with unrestricted supply of water. The experiments were performed following the guidelines set by the Institutional Ethical Committee for animal care and experimentation, College of Pharmacy, University of the Рunjаb, Lаhore, Раkistаn (AEC/PUCP/1094 dated 11 February 2019).

### 4.4. Preparation of the Mucilage 

Mucilage was extracted from dried powdered leaves and fresh fruits following the previously described method by Munir et al. [25]. *Malva parviflora* leaves and fruit mucilage was labelled as MLM and MFM respectively.

### 4.5. Evaluation of the Biological Activities of the Mucilage Extracts

#### 4.5.1. In Vitro Hemolytic Activity

Blood (3 mL) was collected in an anticoagulant tube from a volunteer human (male, 26 years, Blood group O+) by a Phlebotomist in Punjab University Healthcentre lab (Allama Iqbal campus, University of Punjab, Lahore, Pakistan). The sample was centrifuged at 850 rpm for 5 min. The clear supernatant was poured off followed by washing of residue pellets with 5 mL of chilled (4 °C) phosphate buffer saline (PBS) solution (pH 7.4). Washed cells suspension was made in 20 mL cool sterilized PBS. Cells were calculated using hemocytometer and for each assay 7.068 × 10^8^ cell/mL were used. 20 µL of plant mucilage were taken in an Eppendorf tube and PBS and 0.1% TritonX-100 were used as negative and positive control respectively. 180 µL of diluted blood cell suspension were added into each tube followed by incubation at 37 °C. After incubation for 35 min, suspension was allowed to cool for 5 min followed by centrifugation at 1500 rpm for 5 min. 100 µL of supernatant were collected and diluted with 900 µL chilled sterile phosphate buffer saline. An aliquot of 200 µL of all these samples including positive and negative control were transferred to 96-well plate. ELISA microplate reader was used to measure the absorbance at 630 nm [53].

$$\%\;\mathrm{Hemolysis}=\frac{Abs\left(Sample\;absorbance\right)}{Abs\;\left(control\;absorbance\right)}\times 100$$

#### 4.5.2. Acute Toxicity Study

Acute oral toxicity of MLM and MFM was determined in Wistar albino rats according to OECD guidelines 425 [54]. Limit test was performed at 5000 mg/kg where the control group was given only distilled water. The animals were observed for death or any noxious outcome in earliest four hours after the dosing and regularly for fourteen days. During this period, parameters such as weight, physical appearance, behavioral changes, injury, illness signs and mortality were observed. On the 15th day animals were anesthetized and sacrificed with an overdose of xylazine and ketamine anaesthetic drugs and blood samples were obtained by cardiac puncture. Animals were dissected and organs such as liver and kidney were obtained for histopathological examination.

#### 4.5.3. Anti-Inflammatory Activity

Acute anti-inflammatory activity of MLM and MFM was evaluated by carrageenan-induced paw edema in rats [55]. The rats were arbitrarily separated in four groups (*n* = 5). In the first group, the animals received a dose of 10 mL/kg of distilled water (carrageenan control); meanwhile in the second group, the animals were orally administered with diclofenac sodium in a dose of 10 mg/kg (standard group) [56]. In the third and fourth groups, the animals were orally given 500 mg/kg of MLM (MLM experimental group) and MFM (MFM experimental group), respectively. After 1 h, all the animals were injected with 0.1 mL of carrageenan in normal saline (1% (*w*/*v*)) into subplantar region of left hind paw of each rat. Thickness of paw was measured using digital vernier caliper at 0 h, 1 h, 2 h, 3 hand 4 h intervals. Edema inhibition (%) was calculated using the following formula:

$$\%\;\mathrm{inhibition}\;\mathrm{of}\;\mathrm{edema}=\frac{T_{c}-T_{t}}{T_{c}}\;\times \;100$$

where $T_{c}$ = Paw thickness of control group and $T_{t}$ = Paw thickness of experimental group.

#### 4.5.4. Antitussive Activity

Acute antitussive activity of MLM and MFM was evaluated by SO_2_ induced cough model in rats [57]. Each animal served his own control due to variation in number of coughs in each individual animal. Groups were treated in the following manner where in the first group, the animals were orally given codeine phosphate in a dose of 10 mg/kg. In the second and third groups, the animals were orally given 500 mg/kg of MLM and MFM respectively. The animals were exposed to SO_2_ for 60 s after an hour of receiving the treatment and the number of coughs was counted. The number of coughs was compared before and after the treatment to determine the reduction in cough. The percentage inhibition frequency of cough was determined using the following formula:

$$\%\;\mathrm{inhibition}\;\mathrm{of}\;\mathrm{frequency}\;\mathrm{of}\;\mathrm{cough}=\left(\frac{C_{C}-C_{T\;}}{C_{C}}\right)\times 100$$

where $C_{C}$ is the cough frequency in control animal and $C_{T\;}$ is the cough frequency in treated animal [57].

#### 4.5.5. Antiulcer Activity

##### Study Design

The animals were arbitrarily separated into 9 groups (*n* = 6) where the first group (normal group) was orally treated with distilled water (DW) 1 mL/100g [58]. The second group was orally treated with 1 mL/100 g of ethanol (ethanol group) meanwhile from 3–5 groups, the animals received orally standard drugs which are ranitidine (50 mg/kg) [59], omeprazole (20 mg/kg) [60] and sucralfate (100 mg/kg) [61], respectively. Groups 6–7 were orally administered MLM in doses of 250 and 500 mg/kg, respectively whereas groups 8–9 were orally administered MFM in doses of 250 and 500 mg/kg, respectively.

##### Ethanol Induced Acute Gastric Ulcer

Animals were prevented from food for 24 h with unrestricted excess toward water. They were deprived of water just 2 h prior starting the experimental procedure. Animals of all groups were pretreated with drugs and extract as mentioned above. After 30 min all groups were orally administered 90% ethanol (1 mL/100 g) except normal group. Animals were euthanized with an overdose of xylazine and ketamine anaesthetic drugs after 1 h of ethanol administration [62]. Animals were dissected, removed the stomachs and incised along the larger curve. Washed with ice cold normal saline and gastric mucosa was examined [63].

##### Ethanol Induced Chronic Gastric Ulcer

Animals were prevented from food for 24 h with unrestricted excess toward water. They were deprived of water just 2 h prior to starting the experimental procedure. All animals were treated with drug and extract for fourteen days as mentioned above. On the first day of experiment after 30 min above treatment animals were orally administered with 90% ethanol (1 mL/100 g) except normal group. From the 2nd day 30% (*v*/*v*) ethanol was orally given to all groups for fourteen days except normal group. On 14th day, all the animals were anesthetized and sacrificed with an overdose of xylazine and ketamine anaesthetic drugs after 1 h of ethanol administration [62]. Animals were dissected, removed stomachs and incised along larger curve and washed with ice cold normal saline then gastric mucosa was examined [64].

##### Parameters of Gastric Ulcer Evaluation

*Macroscopic and Microscopic Evaluation*

Normal saline was used to wash the stomach and the apparent changes in inner walls of stomach were carefully examined macroscopically by using magnifying glass and microscope. The number of lesions was carefully noted and was used for the determination of ulcer index. Photos were taken using mobile camera (Oppo A57, Guangdong Oppo Mobile Telecommunications Corp., Ltd., Dongguan, Guangdong, China) [65].

##### Ulcer Scoring

Based on severity, the ulcers were given scores as previously reported by Gupta et al. [66] as follows: 0 = no ulcer; 0.5 = reddish mucosa; 1 = red spots; 1.5 = hemorrhagic streaks; 2 = deep ulcers and 3 = perforations.

##### Ulcer Index

Ulcer index can be calculated as previously described by Gul et al. using the following formula [67].

$$\mathrm{Ulcer}\;\mathrm{index}=\left(\mathrm{UN}+\mathrm{US}+\mathrm{UP}\right)\times 10^{-1}$$

where UN = average number of ulcers per animal; US = average of severity score; UP = percentage of animals with ulcer.

##### Ulcer Protection (%)

Ulcer protection (%) was calculated described by Gul et al. using the following formula [67].

$$\%\;\mathrm{Protection}=\frac{\mathrm{Ulcer}\;\mathrm{index}\;\mathrm{of}\;\mathrm{ethanol}\;\mathrm{treated}\;\mathrm{group}-\mathrm{ulcer}\;\mathrm{index}\;\mathrm{of}\;\mathrm{treated}\;\mathrm{group}}{\mathrm{ulcer}\;\mathrm{index}\;\mathrm{of}\;\mathrm{ethanol}\;\mathrm{treated}\;\mathrm{group}\;}\times 100$$

##### Histological Analysis

Samples of stomach walls were fixed in formalin solution (10%) for 48 h and then dehydrated by washing with ascending grades of ethanol. Samples were cleaned by xylene and embedded in paraffin wax. The rotary microtome was used to cut sections of 5–6 mm thickness followed by staining with hematoxylin and eosin. The sections were examined under a microscope for histopathological variations such as ulceration, congestion, edema, necrosis and leukocyte infiltration [68].

##### Mode of Gastro-Protective Activity

*Estimation of the Gastric Volume*

After opening the stomach gastric content was squeezed out in a falcon tube followed by centrifugation at 1000 rpm) for 15 min, then the supernatant was collected, and its volume was determined [69].

##### Determination of the pH Value and Total Acidity

Gastric juice pH was determined using a digital pH meter as previously described by Sen et.al [68]. A 1 mL volume of gastric juice was obtained in a vial then 2 drops of phenolphthalein were added as indicator. Then, it was titrated against 0.1 N NaOH until the end point that is the change from colorless to light pink. The volume of NaOH used was determined then the following formula was used to calculate total acidity where results were expressed in terms of clinical unit mEq/L [70].

$$\mathrm{Total}\;\mathrm{acidity}=\frac{\mathrm{Volume}\;\mathrm{of}\;\mathrm{NaOH}\;\mathrm{used}\times \mathrm{Normality}\;\mathrm{of}\;\mathrm{NaOH}\;\mathrm{used}\times 100}{0.1}$$

##### Determination of Gastric Mucin Content

The glandular portion of stomach was excised and weighed, and this portion was soaked for 2 h in 10 mL of 0.1% Alcian blue solution in 0.16 M sucrose buffered with 0.05 M sodium acetate adjusted to pH = 5. Excess uncomplexed dye was removed by rinsing with 0.25 M sucrose solution at an interval of 15 min and 45 min respectively. Dye forms a complex with mucus that was extracted with 10 mL of 0.5 M magnesium chloride solution for 2 h with consecutive shaking for one minute after 30 min interval. A 4 mL volume of this solution was shaken with an equal volume of diethyl ether. The resulting emulsion was centrifuged at 3000 rpm and the absorbance of aqueous layer was taken at 580 nm. The mucin content of the sample was determined from the standard curve, which was expressed in microgram of Alcian blue extracted per gram of wet gland tissue [71].

##### Determination of the Total Protein Content

Total protein of glandular tissues homogenate was estimated by Lowry method [72]. The tissue homogenate of glandular portion of stomach was mixed with 1 mL of 0.1 M Tris-HCl buffer (pH 7.4) by a homogenizer followed by centrifugation at 1500 rpm for 15 min at 4 °C. The homogenate was diluted with 0.1 M tris-HCl buffer to reach 10 mL. Bovine serum albumin (BSA) was used as standard and 0.1 N of NaOH was used as blank. A 1 mL volume of the sample was mixed with 4.5 mL of reagent I that is formed by mixing 48 mL of 2% Na_2_CO_3_ in 0.1 N NaOH with 1 mL of 1% NaK Tartrate in H_2_O and 1 mL of 0.5% CuSO_4_·5 H_2_O in H_2_O followed by addition of 0.5 mL of reagent II (1 part Folin-Phenol [2 N]: 1 part water) after 10 min. The test tubes were placed in the dark for 30 min and the absorbance was taken at 750 nm. BSA standard calibration curve was used to interpret the results.

### 4.6. Gas Chromatography Coupled with Mass Spectrometry Analysis

Monosaccharide composition of MLM and MFM was analyzed using a modified GC-MS analytical procedure previously adopted by Xia et al. [73] depending upon trimethylsilyl dithioacetal (TMSD) derivatization. A 1 µL volume of the sample was applied to Agilent 7890A Gas Chromatography coupled to Agilent 5975C Mass spectrometer (Agilent Technologies, Santa Clara, CA, USA) with HP-5MS column using a temperature range of 80 °C for 0 min, 80–90 °C at 2.5 °C/min, 190–252 °C at 2 °C/min, 252–300 °C at 25 °C/min, 300–310 °C at 25 °C/min and held for 15 min. Mass spectra were recorded employing total ion chromatogram (TIC) mode and interpreted using NIST 5 software [25].

### 4.7. Statistical Analysis

GraphPad prism 8.4.3 was used for statistical analysis. Statistical significance difference was calculated using one way ANOVA followed by Dunnett’s test. The values *p* < 0.05 *, 0.01 ** and 0.001 *** were considered as statistically significant.

## 5. Conclusions

In conclusion, the current findings suggest that mucilage extracted from the leaves and fruit of *M. parviflora* are safe for in vivo studies and could be incorporated in many significant therapeutic applications after clinical trials. Treatment with mucilage reduced cough, inflammation and ulcers in animal models. The underlying mechanism of gastro protection is in the anti-secretory and mucus protective potential of mucilage. However, further in vivo studies are recommended to comprehensively understand its exact mechanism of action. This study clearly highlights the application of *M. parviflora* mucilage in adjuvant therapy of gastric ulcers. Further studies are in progress to isolate bioactive polysaccharides and glycoprotein from this mucilage.

## Figures and Tables

**Table 1:** Results of biochemical parameters from acute toxicity studies performed using MLM and MFM (5 g/Kg) treated rats.

Groups	Urea (mg/dL)	Creatinine (mg/dL)	Bilirubin (mg/dL)	SGPT (u/L)	SGOT (u/L)	ALP (u/L)
Normal	42.83 ± 1.05	0.9 ± 0.05	0.89 ± 0.039	107.5 ± 9.98	118 ± 8.88	139 ± 6.96
MLM	38.83 ± 1.8 ^ns^	0.82 ± 0.06 ^ns^	0.76 ± 0.05 ^ns^	105.83 ± 11 ^ns^	114.67 ± 7.8 ^ns^	128.5 ± 5.5 ^ns^
MFM	40.5 ± 1.57 ^ns^	0.73 ± 0.05 ^ns^	0.79 ± 0.04 ^ns^	86.67 0± 6.66 ^ns^	114.67 ± 7.8 ^ns^	133 ± 3.21 ^ns^

All values are represented as (Mean ± SEM), ns = not significant.

**Table 2:** Paw thickness at different time intervals in rats treated with MLM and MFM at a dose of 500 mg/kg.

Group Name	Paw Thickness (mm)				
	0 h	1 h	2 h	3 h	4 h
Control	2.09 ± 0.19	3.0 ± 0.16	3.40 ± 0.16	3.52 ± 0.14	3.55 ± 0.15
Standard	2.10 ± 0.26 ^ns^	2.30 ± 0.25 ***	2.38 ± 0.17 ***	2.28 ± 0.20 ***	2.15 ± 0.25 ***
MLM	2.07 ± 0.25 ^ns^	2.68 ± 0.18 *	2.80 ± 0.09 ***	2.71 ± 0.1 ***	2.58 ± 0.17 ***
MFM	2.05 ± 0.16 ^ns^	2.73 ± 0.18 ^ns^	2.99 ± 0.20 **	2.97 ± 0.18 ***	2.99 ± 0.16 ***

Results are expressed as mean ± S.D; Control is distilled water (10 mL/kg); the standard is diclofenac sodium (10 mg/kg). Significant at *p* < 0.05 *, 0.01 ** and 0.001 ***, ns = not significant.

**Table 3:** Effect of MLM and MFM on ulcer score, ulcer index and percentage inhibition in acute ulcer.

Group Name	Ulcer No.	Ulcer Score	Incidence of Ulcer (%)	Ulcer Index	Inhibition of Ulcer (%)
Normal (10 mL/kg p. o)	-	-	-	-	-
Ethanol (10 mL/kg p. o)	7.83 ± 0.60	5.75 ± 0.57	100	11.36	-
Ranitidine (50 mg/kg p. o)	1 ± 0.52 ***	1.0 ± 0.47 ***	50	5.21	54.21
Omeprazole (20 mg/kg p. o)	0.67 ± 0.42 ***	0.58 ± 0.37 ***	33.33	3.46	69.56
Sucralfate (100 mg/kg p. o)	1.16 ± 0.54 ***	1.5 ± 0.67 ***	50	5.27	53.63
MLM (250 mg/kg p. o)	2.0 ± 0.73 ***	2.08 ± 0.69 ***	83.33	8.74	23.04
MLM (500 mg/kg p. o)	1.16 ± 0.60 ***	1.75 ± 0.70 ***	66.66	6.96	38.74
MFM (250 mg/kg p. o)	2.17 ± 0.48 ***	2.5 ± 0.56 **	100	10.47	7.85
MFM (500 mg/kg p. o)	1.5 ± 0.5 ***	2.17 ± 0.70 ***	66.66	7.03	38.08

The results are shown in the form of Mean ± SEM. Significant at *p* < 0.01 ** and 0.001 ***.

**Table 4:** Effect of MLM and MFM on ulcer score, ulcer index and percentage inhibition in chronic ulcer.

Group Name	Ulcer No.	Ulcer Score	Incidence of Ulcer (%)	Ulcer Index	Inhibition of Ulcer (%)
Normal (10 mL/kg p. o)	-	-	-	-	-
Ethanol (10 mL/kg p. o)	3.67 ± 0.71	4.33 ± 0.79	100	10.8	-
Ranitidine (50 mg/kg p. o)	0.67 ± 0.49 ***	1 ± 0.74 ***	33.33	3.5	67.59
Omeprazole (20 mg/kg p. o)	0.83 ± 0.40 ***	1.42 ± 0.70 ***	50	5.22	51.66
Sucralfate (100 mg/kg p. o)	0.83 ± 0.54 ***	0.92 ± 0.58 ***	33.33	3.51	67.5
MLM (250 mg/kg p. o)	1.83 ± 0.79 ***	2.33 ± 1.08 ***	66.67	7.08	34.44
MLM (500 mg/kg p. o)	0.83 ± 0.40 ***	1.58 ± 0.78 ***	50	5.67	47.44
MFM (250 mg/kg p. o)	1.00 ± 3.7 ***	1.83 ± 0.69 **	66.67	6.95	35.65
MFM (500 mg/kg p. o)	0.83 ± 0.54 ***	1.08 ± 0.76 ***	33.33	3.94	63.52

The results are shown in the form of Mean± SEM. Significant at *p* < 0.01 ** and 0.001 ***.

**Table 5:** Effect of MLM and MFM on mucous content and protein content in acute and chronic gastric ulcer.

	Acute Gastric Ulcer		Chronic Gastric Ulcer	
Group Name	Mucous Content	Total Protein	Mucous Content	Total Protein
Normal (10 mL/kg p.o)	482.5 ± 15.59	52.16 ± 3.66	504.17 ± 14.52	74.64 ± 3.71
Ethanol (10 mL/kg p. o)	411.17 ± 9.13	19.55 ± 3.44	295 ± 37.13	38.68 ± 3.26
Ranitidine (50 mg/kg p. o)	452.5 ± 13.14 ^ns^	44.27 ± 3.66 **	445 ± 21.29 ^ns^	72.72± 4.62 ***
Omeprazole (20 mg/kg p. o)	501.67 ± 13.52 ***	38.58 ± 6.37 ^ns^	637.67 ± 32.44 ***	68.41 ± 3.55 ***
Sucralfate (100 mg/kg p. o)	432.5 ± 12.63 ^ns^	28.52 ± 5.93 ^ns^	947.17 ± 65.21 ***	70.46 ± 4.09 ***
MLM (250 mg/kg p. o)	440.5 ± 12.63 ^ns^	23.54 ± 4.46 ^ns^	440.5 ± 12.63 ^ns^	68.58 ± 4.37 ***
MLM (500 mg/kg p. o)	520 ± 17.89 ***	47.47 ± 6.50 **	960.33 ± 58.24 ***	69.35 ± 3.40 ***
MFM (250 mg/kg p. o)	430 ± 12.11 ^ns^	26.73 ± 5.03 ^ns^	840.17 ± 26.24 ***	48.64 ± 3.31 ^ns^
MFM (500 mg/kg p. o)	488.16 ± 17.09 **	30.28 ± 3.95 ^ns^	1239.33 ± 54.29 ***	70.05 ± 4.02 ***

Results are expressed in the form of Mean ± SEM.; Significant at *p* < 0.01 ** and 0.001 ***, ns = not significant; Mucous content are expressed as µg of Alcian blue/g wet tissue; Total protein is expressed as µg/mL.

## Data Availability

Data is contained within the article and Supplementary Materials.

## Supplementary Materials

The following supporting information can be downloaded at: https://www.mdpi.com/article/10.3390/ph15040427/s1, Figure S1: GC/MS chromatograms of neutral (A) and acidic (B) polysaccharides in *M. parviflora mucilage* obtained from the fruits (MFM).
