# Correction: Altyar et al. *Malva parviflora* Leaves and Fruits Mucilage as Natural Sources of Anti-Inflammatory, Antitussive and Gastro-Protective Agents: A Comparative Study Using Rat Models and Gas Chromatography. *Pharmaceuticals* 2022, *15*, 427

**DOI:** 10.3390/ph18050641

**Published:** 2025-04-28

**Authors:** Ahmed E. Altyar, Ans Munir, Saiqa Ishtiaq, Muhammad Rizwan, Khizar Abbas, Osama Kensara, Sameh S. Elhady, Waleed Y. Rizg, Fadia S. Youssef, Mohamed L. Ashour

**Affiliations:** 1 Department of Pharmacy Practice, Faculty of Pharmacy, King Abdulaziz University, P.O. Box 80260, Jeddah 21589, Saudi Arabia; aealtyar@kau.edu.sa; 2 Department of Pharmacognosy, College of Pharmacy, University of the Punjab, Lahore 54000, Pakistan; ansmunir92@gmail.com (A.M.); saiqa.pharmacy@pu.edu.pk (S.I.); 3 Department of Pathology, Lahore Medical and Dental College, University of Health Sciences, Lahore 54600, Pakistan; muhammad.rizwan@lmdc.edu.pk; 4 Department of Pharmacognosy, Faculty of Pharmacy, Bahauddin Zakariya University, Multan 60800, Pakistan; khizarabbas@bzu.edu.pk; 5 Department of Clinical Nutrition, Faculty of Applied Medical Sciences, Umm Al-Qura University, P.O. Box 7067, Makkah 21955, Saudi Arabia; oakensara@uqu.edu.sa; 6 Department of Natural Products, Faculty of Pharmacy, King Abdulaziz University, P.O. Box 80260, Jeddah 21589, Saudi Arabia; ssahmed@kau.edu.sa; 7 Department of Pharmaceutics, Faculty of Pharmacy, King Abdulaziz University, P.O. Box 80260, Jeddah 21589, Saudi Arabia; wrizq@kau.edu.sa; 8 Center of Excellence for Drug Research and Pharmaceutical Industries, King Abdulaziz University, P.O. Box 80200, Jeddah 21589, Saudi Arabia; 9 Department of Pharmacognosy, Faculty of Pharmacy, Ain-Shams University, Abbasia, Cairo 11566, Egypt; fadiayoussef@pharma.asu.edu.eg; 10 Department of Pharmaceutical Sciences, Pharmacy Program, Batterjee Medical College, P.O. Box 6231, Jeddah 21442, Saudi Arabia

## Error in Figure

In the original publication [1], there was a mistake in Figure 2 as published. There was an inappropriate allocation/citation of images in Figure 2. The corrected version of Figure 2 appears below. The authors state that the scientific conclusions are unaffected. This correction was approved by the Academic Editor. The original publication has also been updated.

## Figures and Tables

**Figure 2:**
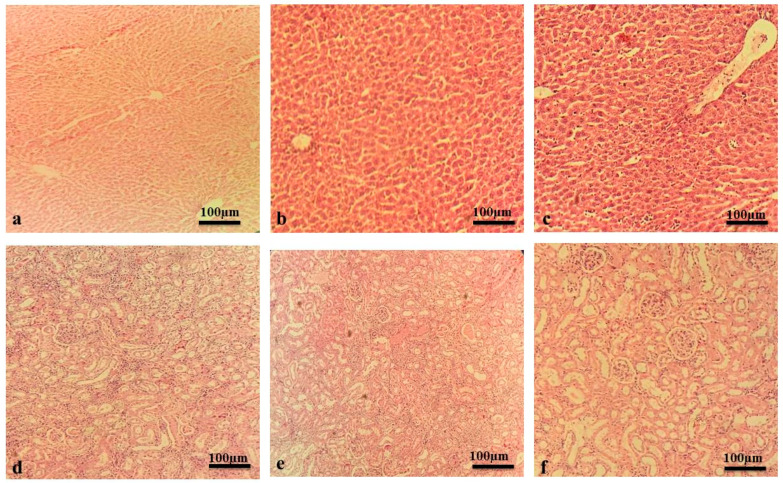
Liver and kidney histology from acute toxicity assay. (**a**) Liver of vehicle control group. (**b**) Liver of MLM-treated group. (**c**) Liver of MFM-treated group. (**d**) Kidney of vehicle control group. (**e**) Kidney of MLM-treated group. (**f**) Kidney of MFM-treated group.
