# Supplementary material for: Malva parviflora Leaves and Fruits Mucilage as Natural Sources of Anti-Inflammatory, Antitussive and Gastro-Protective Agents: A Comparative Study Using Rat Models and Gas Chromatography
Source: Pharmaceuticals (Basel). 2022 Mar 31;15(4):427. doi: 10.3390/ph15040427 (PMC9030788; doi:10.3390/ph15040427)

Malva parviflora **Leaves and Fruits Mucilage as Natural Sources of Anti-inflammatory, Antitussive and Gastro-protective Agents: A Comparative Study Using Rat Models and Gas Chromatography**

Supplementary

Figure S1 GC/MS chromatograms of neutral (A) and acidic (B) polysaccharides in *M. parviflora mucilage* obtained from the fruits (MFM)

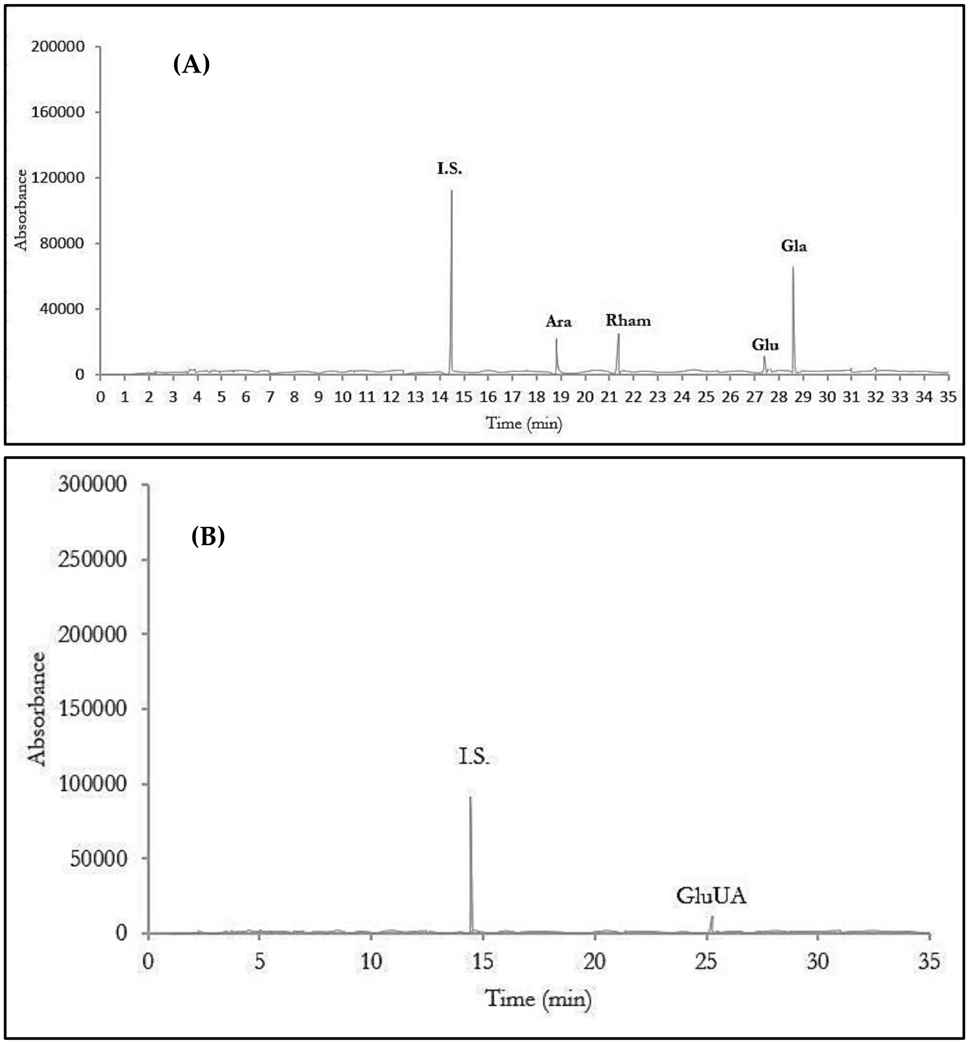

Supplement: Supplementary file 1 [file pharmaceuticals-15-00427-s001.zip › pharmaceuticals-1645049-supplementary.pdf]
